# Identifying risk and protective factors for mental health outcomes: a cross-sectional survey predicting stress, depression, and suicide ideation among Florida farmers

**DOI:** 10.3389/fpubh.2026.1832179

**Published:** 2026-06-29

**Authors:** Carrie N. Baker, Sarah A. Bush, Marshal Sewell, Taylor Sewell, Lauri M. Baker, Sebastian Galindo, Angela B. Lindsey, Christy Chiarelli

**Affiliations:** 1 Department of Agricultural Education and Communication, University of Florida, Gainesville, FL, United States; 2 Mind Your Melon Foundation, Bronson, FL, United States; 3 UF/IFAS Center for Public Issues Education in Agriculture and Natural Resources, Gainesville, FL, United States; 4 Department of Family, Youth, and Community Sciences, University of Florida, Gainesville, FL, United States

**Keywords:** agriculture, occupational health and safety, policy, public health, rural mental health, suicide prevention

## Abstract

**Introduction:**

Due to a variety of personal factors and the nature of their occupation, farmers experience heightened levels of stress that can lead to more severe mental health outcomes. Currently, there is a lack of consistent measures to assess stress and farmer wellbeing nationwide. The purpose of this study was to investigate the mental wellbeing of Florida farmers and identify risk and protective factors for perceived stress, sadness and depression, and suicide ideation.

**Methods:**

This research reports on survey responses from a sample of 373 adult Florida farmers collected from August-November 2024. We used *t*-tests, ANOVAs, chi-square analyses, multiple linear regression, and binary logistic regression to characterize and predict stress, sadness and depression, and suicide ideation using personal and farm-related items.

**Results:**

Farmers reported moderate stress (*M* = 23.61, *SD* = 8.19). *T*-tests and ANOVA results identified stress differences based on gender, marital status, income source, living arrangements, job satisfaction, age, farm role, farm type, and hours worked. The regression model for stress was significant, *F*(26, 259) = 6.939, *p* < 0.001. Age (*p* < 0.001), hours worked (*p* = 0.013), income source (*p* = 0.005), and job satisfaction (*p* < 0.001) predicted farmer stress. Of respondents, 66.9% of farmers reported feeling sad/depressed. Chi square analyses indicated significant differences in sadness/depression based on job satisfaction, farm type, living arrangements, and income source. The logistic regression model for sadness/depression was significant, χ^2^(9) = 136.756, *p* < 0.001. Sadness/depression was predicted by stress (*p* < 0.001), job satisfaction (*p* < 0.001), and farm managerial role (*p* = 0.037). Of respondents, 10.3% of respondents had ideated suicide in the last three months. Chi square analyses indicated differences in suicide ideation for age, relationship status, and job satisfaction. The logistic regression model for suicide ideation was significant, χ^2^(6) = 39.642, *p* < 0.001. Suicide ideation was predicted by stress (*p* < 0.001), age (*p* = 0.007–0.017), and marital status (*p* = 0.003).

**Discussion:**

Findings revealed personal and farming-related factors contributing to stress, sadness/depression, and suicide ideation. This study gathered needed baseline data regarding perceived stress, experiences with sadness and depression, and suicide ideation among Florida farmers that will be helpful for better understanding and, potentially, mitigating risk of Florida farmers' mental health crises in coming years. Recommendations focus on prioritizing prevention over crisis response to target stressors. Findings hold implications for agricultural industry leaders, health professionals, Extension and community educators, and policymakers seeking to address farmer health through legislation, educational programming, and outreach.

## 1 Introduction

Recent attention has identified compounding stressors in agriculture and the impact they are having on the mental health of agricultural producers, their families, and those they employ (1, 2). The American Institute of Stress (3) reports that job stress reached new heights in the years following the COVID-19 pandemic. This finding was further supported by nationwide data indicating that 77% of individuals experience work-related stress (4). When work-related stress is persistent or unchanging, impacts can significantly influence worker health and safety. Farming often blurs the line between livelihood and lifestyle, meaning work demands and the stress that comes from the job is often interwoven with personal challenges.

### 1.1 Stressors of Florida producers

Florida's agricultural industry is critical to the state's economic growth and development. Production agriculture contributes more than $270 billion in revenue and 2 million in jobs to Florida's economy, second only to tourism (5). Across the state, there are approximately 47,500 farms and 9.7 million acres of dedicated farmland, which reap a diverse portfolio of nearly 300 different commodities and agricultural products (5). The state's production is supported by nearly 1,520,938 full- and part-time employees in the agricultural, natural resources, and food (ANRF) industry (5). Not unlike the production portfolio, Florida's agricultural workforce is demographically diverse and responsive to the needs of the state. There are approximately 33,0000 producers, whose primary occupation is farming (6). The average producer in Florida is nearing 59 years of age, with over 11 years of experience on the farm (6). Just over 40% of the state's agricultural producers are female; and while a majority of Florida producers are white, the second largest segment of the workforce is Hispanic, Latino, or Spanish origin (6).

Working in the industry, however, does not come without cost or risk. Florida's agricultural workforce, especially those in underserved communities, face unique challenges and hazards throughout their career, that can have significant impacts on both their physical and mental health (7, 8).

In Florida, over 3.5 million adults live with a mental health condition (9). As research suggests, these conditions can be intensified for those in rural and underserved communities and individuals in high-risk occupations, like agriculture (10, 11). Farmers are exposed and vulnerable to a variety of sociocultural and occupational risk factors that threaten their mental health (12). To many in the agricultural industry, what they do is more than a job or career, but a way of life. Because of this, significant stressors from work infiltrate personal lives and relationships, leading to increased stress, tension, and exhaustion. Most agricultural producers live on their farm and have additional employment off the farm to supplement their income and many work more than 40 h a week (13). In addition to long work hours and a lack of time off the farm, social isolation, family conflict and succession challenges, as well as legislative and regulatory pressures are cited as significant stressors contributing to burnout and hopelessness among farmers (2). Previous research has noted that farmers also perceive the uncertain aspects of their job, such as weather volatility, market fluctuations, taxes, and the many unknowns regarding the future of production agriculture to be “very stressful” (14).

This is especially true in Florida, where extreme weather events and natural disasters like hurricanes, wildfires, heatwaves, and drought often present with very little notice, posing considerable, often life-threatening, damage to workers, crops, livestock, land, and property (15). Natural disaster occurrences and post-crisis response greatly impact the mental health and wellbeing of agricultural workers—often because of the damage these events cause to land, property, or the business at large, which is often generationally-held and intimately connected to family history (16, 17). In Florida, damages caused by Hurricane Irma and the resulting financial and emotional hardship had long-lasting effects on agricultural workers and Extension agents across the state—with reports of felt anxiety increasing nearly a year post-disaster (17).

These stressors (i.e., intensive physical demands from working in agriculture, pressure and uncertainty about the future, financial strain, and conflict between family farm members or business partners) all breed stress and burnout, increase the probability of farm-related injuries, and pose additional hazards to their physical health (18). External stressors are intimately linked to physical symptoms that can occur both from agriculture-related jobs and/or as manifestations of stress (19). For agricultural workers, common symptoms include increased substance use, insomnia, fatigue, weight gain, chest pain, headaches and migraines, heart disease, chronic nerve pain, and high blood pressure (14, 18, 19). These physical symptoms, if untreated can lead to other occupation-related injuries, development or progression of mental illness, self-harm, and thoughts of or completion of suicide (14, 18).

### 1.2 Risk factors for stress and mental disorders

Over the past decade, research on stress, anxiety and depression, and suicide risk among farmers has grown steadily. Previous research indicates stress among farming populations, especially those in the Southeast, is pervasive (18, 20, 21).

Stress is a complex, multifaceted construct shaped by numerous, diverse risk factors. Often these vary by context and population. Frequently cited risk factors for stress in the general population include being female, financial instability, and divorce (22). Other studies have shown inverted U-shaped relationships between age and stress, noting changing associations over time (23).

There are abundant connections in the literature between stress, the compounding effect of stressful life events and more severe mental health outcomes (24). Repeatedly, research indicates higher perceived stress has been associated with emotional distress and depression (25). Studies also associate the experience of stressful life events, burnout, and a lack of perceived job control (26) with suicidal behaviors and ideation.

Empirical evidence also suggests the suicide rate among those working in the agricultural, forestry, fishing, and hunting (AFFH) industries, specifically males, is significantly higher than the general working-age population (11). In a study of Georgia farmers, researchers shared that almost half of their sample experienced sadness or depression and nearly a third ideated suicide in the past year (20).

Research regarding the impact of age on stress and negative mental health outcomes is mixed, though recent research indicates 45% of suicides in the agricultural industry were completed by individuals older than 65 (27).

Through studies that have explored mortality and suicide risk in farming and general populations (28), it is evident there are a variety of risk factors—both demographic and occupation related—that could make farmers vulnerable to stress and mental disorders. In Florida, the state's regulatory environment, inclination to natural disaster, and diverse commodity profile pose potential risks for compounding stress (8). Despite this, Florida lacks baseline data on farmer wellbeing, including stress, depression, and suicide ideation (8).

### 1.3 Purpose and objectives

Therefore, our study aimed to examine mental health outcomes among Florida farmers and to assess associated demographic and farming-related factors, with the intent of obtaining comprehensive baseline data to inform strategic plans for addressing risks and preventing mental health crises in the coming years. Specific objectives were to: (1a) Describe perceived stress in farmers; (1b) Identify significant differences in perceived stress, based on demographics and farming-related factors; (1c) Identify factors predicting perceived stress in farmers; (2) Describe negative emotions experienced by farmers; (3a) Describe experiences with sadness and depression in farmers; (3b) Identify significant differences in mental health outcomes, based on demographics and farming-related factors; (3c) Identify factors predicting sadness and depression in farmers; (4a) Describe suicide ideation in farmers; (4b) Identify significant differences in suicide ideation, based on demographics and farming-related factors; and (4c) Identify factors predicting suicide in farmers.

This study aligns with United Nations Sustainable Development Goal (UN SDGs) Target 3.4, which calls for improved mental health and reduced suicide mortality (29).

Accordingly, it examined key mental health indicators among Florida's farming population and provided actionable recommendations for Mind Your Melon Foundation and Florida Farm Bureau Federation—our non-profit and industry partners—to initiate policy discussions around farmer health and guide strategic, statewide initiatives around mental health education and suicide prevention programming. Beyond our immediate use of results, these findings and recommendations could be relevant for agricultural communicators and industry leaders, public health professionals, Extension educators, and policymakers in Florida and perhaps across the Southeast who are working to address farmer mental health through legislation, education, communication, and community outreach.

## 2 Theoretical framework

We used Lazarus's (30) transactional theory of stress and coping as the theoretical frame. This theory focuses on how individuals assess, perceive, and respond to stressors in their environment (30). Further, it recognizes positive and negative impacts of stress, including the emotional response processes and threat assessment that occurs when presented with a stressor (30). The transactional theory of stress and coping emphasizes the dynamic relationship between stress and coping and proposes when stress outweighs an individuals' ability to cope, the individual experiences threats to their wellbeing and potential negative health outcomes (30). This theory is one of the most widely used theories in stress research and has been applied recently in studies to assess stress, coping, and resilience among farmers (31, 32). During the initial phase—cognitive appraisal of stress—individuals assess their perceived stress or stressors in their life (30). This is a subjective evaluation of stress to gauge threats (30). The initial threat assessment is followed by secondary appraisal, where an individual evaluates the resources they have to overcome or cope with the stressor (30). Our study primarily focused on the appraisal function of the theory, while also considering antecedent conditions—personal and environmental variables—recognized theoretically as factors that impact stress appraisal and response (30). In our study, stress appraisal was operationalized and measured as farmers' perceived stress using the perceived stress scale. This scale has previously been used as a mechanism for appraising farmer stress in studies applying the transactional theory of stress and coping as the theoretical frame (31, 33). We also aimed to identify relationships between stress and more severe negative mental health outcomes—experiences with sadness and depression and suicide ideation—given their proposed relationship in the theory (30). Lazarus (30) posit that early appraisal and stress awareness promotes stress resilience and allows for optimum stress response. We sought to examine how Florida farmers appraise or perceive their stress and identify personal and role-related antecedents that could impact their ability to cope.

## 3 Materials and methods

### 3.1 Study design and ethics

This manuscript reports on data that was part of a larger study using cross-sectional survey design to examine the mental wellbeing of Florida's agricultural industry. For the larger study, we worked with representatives from Mind Your Melon Foundation, a non-profit focused on agricultural health and wellbeing and the Florida Farm Bureau Federation to design and implement the Mind Your Melon Farmer Wellbeing Survey. This comprehensive survey gathered data on mental and physical health, care access, and mental health help-seeking preferences of individuals involved in Florida's agricultural industry (e.g., farmers, farm spouses, industry and Extension professionals). This manuscript focuses specifically on risk and protective factors for mental health outcomes for a farmer-only sample and is the first manuscript of this study to be published. Participants provided their informed consent to participate in the broader study, which was approved by University of Florida Institutional Review Board (UFIRB) as exempt under #IRB202400939. These community partners completed research ethics training to become co-investigators through the academic institution.

### 3.2 Sampling and data collection procedures

We collected data from August-November 2024 using participatory protocols co-created with our community partners. Individuals involved in Florida's agricultural industry over the age of 18, including farmers, spouses, and industry and Extension professionals were invited to participate. Those who did not meet this criteria were excluded. Florida is diverse agriculturally, producing between 200 and 300 commodities across 67 counties (34). To facilitate broader statewide participation, we used a combination of non-probability sampling techniques including in-person and online recruitment, as random sampling was not feasible (35).

We partnered with Florida Farm Bureau Federation and a network of more than 40 major agricultural associations, commodity groups, and Extension offices to distribute the survey through an online link via email and social media. We aimed to maximize the survey's reach and recruitment potential for practical utility for our non-profit and community partners, while maintaining academic rigor. To help achieve this, we co-created a recruitment protocol and toolkit (e.g., approved media, distribution assets, templated messaging, timeline of survey distribution and reminders) with instructions for sharing the survey with their membership and stakeholders in ways that adhered to Dillman's tailored design method for survey research (36).

The survey launched online in early August 2024 with a priming email and press release from Florida Farm Bureau Federation followed later by an initial email with the survey link. In line with Dillman et al. (36) Florida Farm Bureau Federation sent two reminder emails before the survey closed on November 10. These email notices coincided with social media recruitment. Agricultural industry partners were asked to adhere to the survey recruitment protocol using materials provided in the toolkit.

To facilitate broader statewide participation, our research team also attended seven annual meetings during the data collection window and employed a modified venue-based sampling approach, which is cited as effective for hard-to-reach populations, like farmers (37). At these events, participants were provided with a QR code to the survey link and iPads with surveys were available if they wished to complete the survey on site (36).

### 3.3 Instrumentation and measurement

Our online, Qualtrics-based survey was an adaptation of a previously piloted, validated survey developed by Mercer University School of Medicine researchers to address the mental wellbeing of Georgia farmers (20, 38–42). This was, in part, to support continued validation of the instrument for future replication and cross-state comparisons. Our survey instrument consisted of pre-existing, validated instruments, including the perceived stress scale (PSS) (43, 44) and additional items from the previous pilot survey including personal and farming-related demographics and items pertaining to stress and mental health outcomes, like anxiousness and worry (20, 38–42). The survey, which was developed in English, took participants approximately 20 min to complete. Given the demographic makeup of Florida's agricultural workforce, a translator made the survey accessible to Spanish speakers. The instrument was reviewed by a panel of academic and industry experts for face and content validity (36).

#### 3.3.1 Personal and farming-related demographics

We asked participants a series of demographic items regarding their age, gender, race/ethnicity, education, dependents, relationship status, county of residence, and household income. Other demographic items regarding finances and family were asked but not included in this analysis. Farming-related demographics included industry or farming role, work schedule, first-generation designation, farm living arrangements, and farm size. We used census-based demographic items from the Qualtrics certified library (45), demographic items from the previous pilot survey (20), and additional farming items after input from the expert panel (36). Farm size was based on the U.S. Department of Agriculture Economics Research Service (USDA-ERS) farm typology (46).

#### 3.3.2 Perceived stress

The PSS (43) is one of the most widely known, accepted measures of stress among health scholars, practitioners, and social scientists. Extensive literature supports the psychometric rigor of the PSS including its factor structure (47), concurrent, and predictive validity (43, 48) and reliability. While shorter versions are available, we chose to use the 14-item version. Researchers continue to use PSS-14 (49, 50) as a way for individuals to characterize the degree to which they appraise their current life situation as stressful (44). More recently, the PSS has been used with farming populations to assess and investigate associated factors (12, 51, 52). The PSS asks about how often individuals experience certain feelings, we specified within the past 3 months. Respondents rate items on a five-point Likert-type scale (0 = *Never*, 1 = *Almost Never*, 2 = *Sometimes*, 3 = *Fairly Often*, 4 = *Very Often*). Positively worded items were reverse coded and PSS scores were obtained by summing all scale items that could range from 0 to 56 (43). Multiple studies have confirmed PSS-14 demonstrated adequate reliability (α = 0.75–0.91) (47). A *post hoc* reliability assessment confirmed adequate internal consistency of the PSS in our study (α = 0.88).

#### 3.3.3 Negative emotions, mental health outcomes, and suicide ideation

We asked nine items to encompass negative emotions or mental health outcomes individuals in Florida's agricultural industry might experience. Participants were asked how often they experienced the following negative emotions within the past 3 months: (a) feeling unhappy in their current role; (b) scared that you would lose your job; (c) Scared that you would lose a significant amount of income; (d) scared that you would lose your farm (farm owner/farm spouse only); (e) feeling nervous or worried about the future; (f) feeling lonely; (g) feeling nervous or worried about the future; (h) feeling sad or depressed; (i) feeling hopeless or like you will never be happy; and (j) thoughts of wanting to die by suicide. Participants responded to items on a five-point Likert scale (0 = *Never*, 1 = *Less than once per month*, 2 = *At least once per month*, 3 = *At least once per week*, 4 = *Daily*).

We used these individual items to create binary variables for “sadness or depression” (Yes/No) and “suicide ideation” (Yes/No). Feelings of sadness and depression were classified as a “yes” if participants indicated they felt sad or depressed at least once per month within the past 3 months (i.e., *at least once per month, at least once per week*, or *daily* was selected). Suicide ideation was classified as a “yes” if participants said they had thoughts of suicide within the past 3 months (i.e., any item besides *never* was selected). This approach was consistent with the previously piloted Georgia study from which this study was adapted (20, 42).

Participants were shown a 911 public service announcement (PSA) and the 988 Suicide and Crisis Hotline (See Supplementary material S1) following questions about feeling hopeless, sad or depressed, and suicide ideation if they indicated experiencing these feelings repeatedly. At the request of the IRB office, we also provided a text entry for participants who requested to be contacted and connected with a local mental health professional or resources.

### 3.4 Data cleaning and analysis

The lead author transferred data to IBM SPSS Statistics Version 29.0 (IBM Corp.) and removed blank responses prior to analysis. As previously mentioned, this data was collected as part of the Mind Your Melon Farmer Wellbeing Survey (*N* = 671), which surveyed adults involved in Florida's agricultural industry. However, this manuscript reports on results from a farmer-only subsample—respondents whose primary occupation was farming. We had 373 farmer responses. Partial and missing responses were handled by case using listwise deletion in SPSS.

We used frequencies and percentages, means, and standard deviations to characterize demographic information and describe the perceived stress, negative emotions, sadness and depression, and suicide ideation among farmers.

We used independent samples *t*-tests, one-way Analysis of Variance (ANOVAs), and Chi square analyses, as appropriate, to conduct group comparisons and assess whether perceived stress, experiences of sadness and depression, and suicide ideation differed by demographic and farming-related factors. We followed up significant ANOVAs with Bonferroni *post hoc* tests to control for Type 1 errors. Bonferroni-adjusted *p* values are reported for pairwise comparisons.

Finally, we used regression analyses to identify demographic and farming-related factors that predict mental health outcomes. Multiple linear regression was used to model predictors of perceived stress and binary logistic regression was used to model predictors of sadness and depression and suicide ideation.

## 4 Results

This manuscript reports on results as part of a larger research study. Of the 373 farmers, respondents identified their primary role as either farm owner (*n* = 294, 78.8%), farm manager/supervisor (*n* = 58, 15.6%), or farmworker (*n* = 21, 5.6%). This paper focuses on the farmer-only sample. We had varying levels of response to demographic items and percentages reflect those differences.

### 4.1 Participants

The majority of farmers were white (*n* = 348, 94.5%), married (*n* = 267, 77.4%), males (*n* = 221, 61.2%), without children in the household (*n* = 183, 52.4%), between 35 and 54 years old (*n* = 84, 40.6%). Our sample was made up of primarily English-speaking (*n* = 286, 96.6%), U.S. citizens (*n* = 287, 98.3%. Our sample was highly educated, with majority holding a bachelor's (*n* = 136, 39.0%) or graduate/professional degree (*n* = 78, 22.3%). The greatest proportion reported a household income of $200,000 or more (*n* = 93, 26.6%; See Table 1).

**Table 1:** Participant demographics (*n* = 373).

Demographics	*N*	*f*	Valid %
Gender	361		
Male		221	61.2
Female		138	38.2
Prefer not to say		1	0.3
Prefer to self-describe		1	0.3
Age	207		
18–34 years		39	18.8
35–54 years		84	40.6
55–74 years		73	35.3
75 and older		11	5.3
Race/Ethnicity	368		
White or Caucasian		348	94.5
Hispanic/Latino/Spanish		11	3.0
American Indian/Native American or Alaska Native		5	1.4
Asian		3	0.8
Black or African American		1	0.3
Marital status	345		
Single, never married		35	10.1
Living with partner		12	3.5
Married		267	77.4
Divorced/Separated		19	5.5
Widowed		12	3.5
Do children live in your household?	349		
No		183	52.4
Yes		165	47.3
Prefer not to answer		1	0.3
Highest level of education	349		
Some high school or less		3	0.9
High school diploma or GED		33	9.5
Some college, but no degree		59	16.9
Associates or technical degree		38	10.9
Bachelor's degree		136	39.0
Graduate or professional degree		78	22.3
Prefer not to say		2	0.6
Household income	350		
Less than $25,000		10	2.9
$25,000–$49,999		22	6.3
$50,000–$74,999		42	12.0
$75,000–$99,000		46	13.1
$100,000–$149,000		83	23.7
$150,000–$199,000		54	15.4
$200,000 or more		93	26.6
Primary/First language	296		
English		286	96.6
Portuguese		3	1.0
Spanish		7	2.4
Residential status	292		
U.S. citizen		287	98.3
Legal permanent resident		2	0.7
Prefer not to answer		3	1.0

The largest percentage of farmers lived in the Northeast region (*n* = 79, 30.5%) and reported the farm as their primary residence year-round (*n* = 212, 61.5%). The greatest percentage of respondents farmed full-time (*n* = 211, 57.2%) on small family farms (*n* = 172, 51.3%) but did not rely on the farm as their family's sole income source (*n* = 258, 72.1%). Respondents most often reported working approximately 41–60 h a week (*n* = 104, 31.2%) during busy season. Most had generational farming backgrounds (*n* = 240, 69.6%) and had been farming between 21 and 40 years (*n* = 107, 31%; see Table 2).

**Table 2:** Farming characteristics of participants (*n* = 373).

Demographics	*N*	*f*	Valid %
Primary role	373		
Farm owner		294	78.8
Farm manager or supervisor		58	15.5
Farmworker or hired worker		21	5.6
Classification of work as a farmer	369		
Full-time		211	57.2
Part-time		109	29.5
Hobby		43	11.7
Retired		6	1.6
Farming background	345		
First-generation		105	30.4
Generational		240	69.6
Income source	358		
Other income sources		258	72.1
Farm-only		94	26.3
Prefer not to answer		6	1.7
Years farming	345		
Less than 1 year		5	1.4
1–5 years		38	11.0
6–10 years		38	11.0
11–20 years		73	21.2
21–40 years		107	31.0
41–50 years		43	12.5
More than 50 years		41	11.9
Farm size—USDA designation^a^	336		
Small family farm (GCFI, less than $350K)		172	51.3
Midsize family farm (GCFI, $350K−999,999K)		54	16.1
Large family farm (GCFI of $1M or more)		77	23.0
Non-family farm		32	9.6
Living arrangements	344		
Live on farm year-round		212	61.6
Live on farm part-time		13	3.8
Live off farm		119	34.6
Busy season—Hours worked	333		
0–20 h		40	12.0
21–40 h		49	14.7
41–60 h		104	31.2
61–80 h		92	27.6
81–100 h		43	12.9
More than 100 h per week		5	1.5
State region by extension district	259		
Northeast		79	30.5
Southeast		57	22.0
Southwest		56	21.6
Central		48	18.5
Northwest		19	7.3

^a^Farm size was based on the farm they own/manage/work on. Non-family farms were defined as those where any operator or individual related to respondent does not own a majority (50%) of the business.

Over 40 state associations and at least 20 different industry or commodity sectors (see Table 3) were represented in our sample. Beef production (*f* = 178) was the most represented sector.

**Table 3:** Industry sector or commodities represented across sample (*n* = 373).

Industry sector or commodity	*f*	%
Beef	178	47.7
Hay and forage	89	23.9
Other fruits and vegetables	81	21.7
Citrus	50	13.4
Timber	49	13.1
Nursery and landscape	26	7.0
Equine	25	6.7
Sod/turfgrass	22	5.9
Peanuts	20	5.4
Other produce/crops	20	5.4
Eggs	19	5.1
Other livestock	17	4.6
Wheat, corn, and other grains	13	3.5
Other allied industry	13	3.5
Apiculture	12	3.2
Dairy	12	3.2
Poultry	9	2.4
Sugar	8	2.1
Cotton	6	1.6
Aquaculture	5	1.3

Examples of other produce and/or crops reported by farmers were: blueberries, watermelons, strawberries, grapes, peaches, microgreens, peppers, tropical fruit and/or nut trees, avocadoes, potatoes, cover crops, fruit and nut trees, flowers and foliage. Examples of other livestock reported by farmers were: goats, swine, rabbits, and sheep. Examples of other allied industries reported by farmers mostly pertained to agritourism, agricultural education, rescue farms and sanctuaries, conservation and wildlife management, and other custom services.

### 4.2 Perceived stress among Florida farmers

#### 4.2.1 Objective 1a: describe perceived stress

Florida farmers reported moderate levels of perceived stress (*M* = 23.61, *SD* = 8.19), where the mean represented the sum of scale items calculated and averaged across participants who responded to the scale in full (*n* = 310). The item with the highest mean was, “Found yourself thinking about things you have to accomplish” (*M* = 3.17, *SD* = 0.80), followed by “Felt nervous and ‘stressed”' (*M* = 2.21, *SD* = 1.16; see Table 4).

**Table 4:** Means and standard deviations for perceived stress scale (PSS-14) items.

Items	*N*	*M*	SD
Been upset because of something that happened unexpectedly	313	1.78	0.93
Felt that you can't control the important things in your life	313	1.55	1.12
Felt nervous and “stressed”	312	2.21	1.16
Felt that you dealt successfully with annoying life problems^a^	312	1.33	0.87
Felt that you were handling important changes in your life well^a^	311	1.28	0.80
Felt confident about your ability to handle personal problems^a^	312	1.01	0.78
Felt that things were going the way you wanted them to go^a^	312	1.45	0.84
Found that you could not cope with all the things you had to do	312	1.46	1.01
Been able to control irritations in your life^a^	312	1.39	0.89
Felt that you were getting everything done that you needed to get done^a^	312	1.94	1.00
Been angered because of things that happened you could not control	313	1.94	0.91
Found yourself thinking about things that you have to accomplish	313	3.17	0.80
Been able to control the way you spend your time^a^	313	1.53	0.82
Felt difficulties piling were up so high you could not overcome them	313	1.56	1.01

^a^Reversed scored item. Participants were asked to indicate how often they experienced these thoughts or feelings in the past 3 months.

#### 4.2.2 Objective 1b: identify significant differences in perceived stress

We then sought to identify if there were significant differences in farmers' PSS scores based on certain demographic and farming-related factors using *t*-tests (see Table 5) and ANOVAs (see Table 6). *T*-tests indicated stress was higher among females (*p* = 0.002), unmarried individuals (*p* = 0.012), those with farm-only income (*p* < 0.001), living off the farm (*p* = 0.038), and frequently unhappy in their role (*p* = < 0.001).

**Table 5:** Independent *T*-test results comparing PSS scores of Florida farmers by dichotomous factors.

Variable	*N*	PSS Score		*t* (df)	*p-*Value	*d*
		*M*	SD			
Gender						
Male	195	22.49	8.17	−3.12 (306)	0.002^**^	0.369
Female	113	25.46	7.83			
Married or live-in partner						
Yes	248	22.93	8.29	−2.533 (304)	0.012^*^	0.369
No	58	25.91	6.98			
Children in home						
Yes	148	24.41	7.68	1.654 (308)	0.099	0.188
No	162	22.88	8.58			
Generation farmer						
First-gen	94	23.27	8.40	−0.487 (308)	0.627	0.060
Generational	216	23.76	8.11			
Income source						
Farm-only	82	27.20	7.64	4.785 (308)	< 0.001^***^	0.616
Other	228	22.32	8.01			
Living arrangements						
On-farm	197	22.93	8.16	−2.08 (307)	0.038^*^	0.246
Off-farm	112	24.93	8.05			
Unhappy with role^a^						
Frequently	122	28.68	6.52	10.124 (308)	< 0.001^***^	1.18
Never or rarely	188	20.32	7.46			

Never or rarely (never or less than once per month), Frequently (At least once per month, at least once per week, or daily).

^*^,^**^,^***^ denotes significance at 0.05, 0.01, and 0.001 levels, respectively.

**Table 6:** ANOVA results comparing differences in PSS scores across categories of multilevel predictors.

Variable	*N*	PSS Score		*t* (df)	*p-*Value	*d*
		*M*	SD			
Age						
18–34 years	33	27.21	6.72	10.910 (3, 175)	< 0.001^***^	0.158
35–54 years	76	23.59	6.87			
55–74 years	61	19.13	9.09			
75 and older	9	15.67	6.98			
Education						
HS, up to some college	78	23.55	7.68	0.310 (3, 305)	0.818	0.003
Associates or technical	36	23.72	8.41			
Bachelor's degree	123	24.02	8.53			
Graduate or professional	72	22.85	8.16			
Income						
Less than $100,000	104	24.78	7.51	1.489 (2, 302)	0.227	0.010
$100,000–$199,000	119	23.05	8.84			
$200,000 or more	82	23.10	8.08			
Relationship status						
Single, never married	32	25.38	6.06	2.232 (4, 301)	0.066	0.029
Living with partner	9	23.89	10.96			
Married	239	22.90	8.20			
Divorced or separated	17	28.24	8.43			
Widowed	9	23.44	6.56			
Primary farming role						
Farm owner	243	22.82	8.12	5.614 (2, 307)	0.004^**^	0.035
Farm manager	50	26.04	7.65			
Farmworker	17	27.71	8.51			
Farm type						
Small family	154	21.72	8.00	6.241 (3, 302)	< 0.001^***^	0.058
Midsize family	48	25.42	7.18			
Large family	74	25.73	8.04			
Non-family	30	25.83	8.67			
Weekly hours^a^						
0–20 h	34	19.79	8.84	5.872 (5, 295)	< 0.001^***^	0.091
21–40 h	41	19.32	7.59			
41–60 h	94	23.74	7.67			
61–80 h	89	25.33	8.37			
81–100 h	39	25.56	6.94			
100+ h	4	30.25	5.74			

^a^During busy season.

^**^,^***^ denotes significance at 0.05, 0.01, and 0.001 levels, respectively.

We visually inspected a Q-Q plot and performed a Shapiro–Wilk test, which indicated PSS was normally distributed, *W*(310) = 0.992, *p* = 0.117. Levene's test was used to evaluate if the assumption of homogeneity of variance was met.

One-way ANOVA results identified significant difference in stress between age groups (*F*_(3, 175)_ = 10.910, *p* = < 0.001), farm roles (*F*_(2, 307)_ = 5.614, *p* = 0.004), farm type (*F*_(3, 302)_ = 6.241, *p* = < 0.001), and weekly hours worked (*F*_(5, 295)_ = 5.872, *p* < 0.001). Bonferroni-corrected *post hoc* tests indicated stress among those aged 18–34 (*M* = 27.21, *SD* = 6.72) was significantly higher than those aged 55–74 (*M* = 19.13, *SD* = 9.09, *p* < 0.001) and those 75 and older (*M* = 15.67, *SD* = 6.98, *p* < 0.001). Similarly, stress was higher among those aged 35–54 (*M* = 23.59, *SD* = 6.87) relative to farmers 55–74 years (*p* = 0.005) and 75 years and older (*p* = 0.023). Stress among farmworkers (*M* = 27.71, *SD* = 8.51) was significantly higher than farm owners (*M* = 22.82, *SD* = 8.12, *p* = 0.049). Stress among farm managers/supervisors (*M* = 26.04, *SD* = 7.65) was significantly higher than farm owners (*p* = 0.032). Regarding farm type, stress was significantly lower for those on small family farms (*M* = 21.72, *SD* = 8.00), relative to those on midsize family (*M* = 25.42, *SD* = 7.18, *p* = 0.032), and large family (*M* = 25.73, *SD* = 8.04, *p* = 0.003). Those who worked 0–20 weekly hours (*M* = 19.79, *SD* = 8.84) reported significantly less stress relative to those working overtime at 61–80 h (*M* = 25.33, *SD* = 8.37, *p* = 0.009) and 81–100 h (*M* = 25.56, *SD* = 6.94, *p* = 0.031). Similarly, those working part-time at 21–40 h (*M* = 19.32, *SD* = 7.59) also reported significantly less stress relative to those working 41–60 h (*M* = 23.74, *SD* = 7.67, *p* = 0.045), 61–80 h (*p* = 0.001), and 81–100 h (*p* = 0.007).

#### 4.2.3 Objective 1c: predict stress

To identify predictive stress factors, we first checked to ensure the data did not violate regression assumptions. The Q-Q plot supported normality and VIF values less than 2 indicated no significant signs of multicollinearity. Durbin-Watson statistic (*DW* = 2.18) fell within the acceptable range, suggesting independence of errors assumption had been met. Finally, an insignificant Breusch–Pagan test χ^2^ = 0.755, *p* = 0.796, indicated the assumption of homogeneity of variance was met.

Our regression model using gender, age, relationship status, children in the home, education level, income, first-gen status, primary farming role, income source, job satisfaction, living arrangements, farm type, and weekly hours as predictors explained a significant amount of variation in Florida farmers' (*n* = 286) perceived stress, *F*_(26, 259)_ = 6.939, *p* < 0.001, *R*^2^ = 0.411. Accounting for the number of predictors, the adjusted percentage of variance explained was 35.1%. Model results indicated age (*p* < 0.001), relationship status (*p* = 0.016), income source (*p* = 0.005), job satisfaction (*p* < 0.001), and weekly hours worked (*p* = 0.013) were significant predictors of perceived stress scores in Florida farmers (see Table 7).

**Table 7:** Regression coefficients from the MLR model predicting stress among Florida farmers (*n* = 286).

Variable	*B (S.E)*	*t*	*p-*Value	β
Intercept	20.891 (1.694)	12.329	< 0.001^***^	–
Gender (Male)	−2.368 (0.933)	−2.537	0.012^*^	−0.140
Age	−0.003 (0.001)	−3.759	< 0.001^***^	−0.191
Relationship status				
Single, never married	−0.059 (1.583)	−0.037	0.970	−0.002
Living with partner	1.551 (2.657)	0.584	0.560	0.029
Divorced or separated	4.550 (1.879)	2.421	0.016^*^	0.121
Widowed	0.527 (2.633)	0.200	0.842	0.011
Children (Yes)	−0.171 (0.919)	−0.186	0.852	−0.011
Education				
HS, up to some college	0.307 (1.042)	0.294	0.769	0.016
Associates or technical	1.130 (1.337)	0.845	0.399	0.046
Graduate or professional	−0.166 (1.070)	−0.155	0.877	−0.009
Income				
$100,000–$199,000	−0.103 (1.018)	−0.101	0.920	−0.006
$200,000 or more	0.635 (1.199)	0.529	0.597	0.035
First-gen farmer	0.355 (0.953)	0.373	0.709	0.020
Primary farming role				
Farm manager	−0.484 (1.280)	−0.378	0.706	−0.022
Farm worker	3.427 (2.133)	1.607	0.109	0.091
Income source (Farm-only)	2.968 (1.057)	2.808	0.005^*^	0.163
Job satisfaction (Unhappy)	6.963 (0.870)	8.001	< 0.001^***^	0.419
Living arrangements (On-Farm)	−1.548 (0.926)	−1.671	0.096	−0.092
Farm type				
Midsize family	007 (1.298)	0.005	0.996	0.000
Large family	−0.787 (1.286)	−0.612	0.541	−0.041
Non-family	0.275 (1.768)	0.156	0.876	−0.010
Weekly hours^a^				
0–20 h	−1.591 (1.527)	−1.042	0.298	−0.060
21–40 h	−3.387 (1.354)	−2.502	0.013^*^	−0.146
61–80 h	1.057 (1.056)	1.001	0.318	0.060
81–100 h	1.394 (1.365)	1.021	0.308	0.058
100+ h	5.095 (3.462)	1.472	0.142	0.074

^a^Based on busy season. Categorical variables were dummy coded. The reference group for gender was female. The reference group for relationship status was married. The reference group for children was no. The reference group for education was bachelor's degree. The reference group for income was Less than $100,000. The reference group for First-Gen was generational farmer. The reference group for primary farm role was farm owner. The reference group for income source was other sources of income. The reference group for living arrangements was off-farm. The reference group for farm type was small family. The reference group for weekly hours was 41–60 h.

Holding all other variables constant, increase in age was associated with an average, statistically significant decrease in perceived stress score (*t* = −3.759, *p* < 0.001). Perceived stress for divorced or separated farmers was statistically significantly higher than married farmers by 4.55 (*t* = 2.421, *p* = 0.016). Those with farm-only income had significantly higher perceived stress than farmers with other income streams by 2.968 (*t* = 2.808, *p* = 0.005); and those who indicated they were frequently unhappy with their role had significantly higher perceived stress than those who were satisfied in their role by 6.963 (*t* = 8.001, *p* < 0.001). Finally, farmers who worked part-time at 21–40 h reported significantly less stress than those working on average 41–60 h a week by 3.837 (*t* = −2.502, *p* = 0.013).

### 4.3 Objective 2: describe negative emotions

Item means for negative emotions fell between response items 0 (*Never*) and close to 2 (*at least once per month*), indicating participants experienced these emotions in the past 3 months, but not overly frequently (see Table 8). Farmers indicated in the 3 months prior to taking the survey, the most frequent role-related negative emotions they experienced was feeling scared they would lose a significant amount of income (*n* = 328, *M* = 1.53, SD = 1.27). Findings indicated 78.3% of respondents worry *at least monthly* about income loss—with 9.8% of those indicating they worry *daily* about income. Farmers less frequently reported they were unhappy in their roles (*n* = 330, *M* = 1.19, *SD* = 1.19). Majority of respondents (*f* = 200. 60.6%) indicated they were rarely (*never* or *less than once per month*) unhappy in their roles. Similarly, farmers were less concerned about losing their farm (*n* = 265, *M* = 1.02, *SD* = 1.21) or their job (*n* = 330, M = 0.75, SD = 1.11).

**Table 8:** Frequency of role-related negative emotions experienced by Florida farmers (*n* = 373) within the past 3 months.

Items	*N*	*f*	%
Scared you would lose significant income	328		
Never		88	26.8
Less than once per month		80	24.4
At least once per month		89	27.1
At least once per week		39	11.9
Daily		32	9.8
Feeling unhappy with role	330		
Never		132	40.0
Less than once per month		68	20.6
At least once per month		77	23.3
At least once per week		41	12.4
Daily		12	3.6
Scared you would lose your farm^a^	265		
Never		120	45.3
Less than once per month		74	27.9
At least once per month		35	13.2
At least once per week		18	6.8
Daily		18	6.8
Scared you would lose your job	330		
Never		197	59.7
Less than once per month		62	18.8
At least once per month		38	11.5
At least once per week		21	6.4
Daily		12	3.6

^a^Item only shown to individuals whose primary role = farm owner.

Anxious thoughts were most prevalent (*n* = 329, *M* = 2.12, *SD* = 1.28), with 64.4% (*n* = 329) of farmers indicating they felt nervous or worried about the future at least once a month or more in the past 3 months. Less often farmers felt lonely (*n* = 330, *M* = 1.02, *SD* = 1.21) and hopeless (*n* = 329, *M* = 0.73, *SD* = 0.5). Though, 30.3% of respondents still indicated feeling lonely at least once a month or more over the past 3 months; and 21.3% indicated feeling hopeless at the same rate (see Table 9).

**Table 9:** Frequency of mental health outcomes experienced by Florida farmers (*n* = 373) within the past 3 months.

Items	*N*	*f*	%
Feeling nervous or worried about the future	329		
Never		40	12.2
Less than once per month		77	23.4
At least once per month		71	21.6
At least once per week		85	25.8
Daily		56	17.0
Feeling sad or depressed	330		
Never		109	33.0
Less than once per month		106	32.1
At least once per month		64	19.4
At least once per week		37	11.2
Daily		14	4.2
Feeling lonely	330		
Never		159	48.2
Less than once per month		71	21.5
At least once per month		52	15.8
At least once per week		32	9.7
Daily		16	4.8
Feeling hopeless or like you will never be happy	329		
Never		196	59.6
Less than once per month		63	19.1
At least once per month		41	12.5
At least once per week		20	6.1
Daily		9	2.7
Thoughts of wanting to die by suicide	330		
Never		296	89.7
Less than once per month		26	7.9
At least once per month		3	0.9
At least once per week		4	1.2
Daily		1	0.3

Participants were shown a 911 PSA and the 988 Suicide and Crisis Hotline following questions about feeling hopelessness, sadness or depression, and suicide ideation if they indicated experiencing these feelings. At the request of the UF IRB, we also provided a text entry for participants who requested to be contacted and connected with a local mental health professional or resources.

### 4.4 Sadness and depression among Florida farmers

#### 4.4.1 Objective 3a: describe sadness and depression

After anxiousness, the next most frequent emotion experienced by farmers was sadness or depression (*n* = 330, *M* = 1.22, *SD* = 1.15). Of farmers who responded results indicated that 66.9% reported feeling sad or depressed at some point in the past 3 months. Of those, 32.1% experienced sadness or depression less than once per month, 19.4% at least once a month, 11.2% at least once a week, and 4.2% felt sad or depressed daily (see Table 9). These item responses were transformed into event counts for a binary variable. Those who indicated that in the past 3 months they felt sad or depressed at least once per month or more were coded as a “Yes” (*n* = 115). All others were coded as a “No” (*n* = 215).

#### 4.4.2 Objective 3b: identify significant differences in sadness and depression

We aimed to identify if there were significant differences in experiencing sadness and depression based on personal demographics and farm-related factors. Chi-square analyses indicated significant relationships between experiencing sadness/depression and job satisfaction, χ^2^ (1, *N* = 330) = 71.235, *p* < 0.001, ϕ = 0.465; farm type χ^2^ (3, *N* = 325) = 13.213, *p* = 0.004, ϕ = 0.202; living arrangements χ^2^ (1, *N* = 329) = 6.399, *p* = 0.011, ϕ = 0.139), and income source χ^2^ (1, *N* = 330) = 9.733, *p* = 0.002, ϕ = 0.172; see Table 10).

**Table 10:** Identifying differences in farmers' experience with sadness and depression based on demographic and farming-related factors.

Variable	*N*		*χ^2^*	df	*p-*Value	ϕ
	Yes (*n* = 115)	No (*n* = 215)				
Gender (*n* = 328)						
Male	68	140	0.779	1	0.377	0.049 ^a^
Female	45	75				
Age (*n* = 190)						
18–34 years	15	19	5.678	3	0.128	0.128^c^
35–54 years	31	47				
55–74 years	16	51				
75 and older	4	7				
Education (*n* = 329)						
HS, up to some college	29	57	0.096	3	0.992	0.017^c^
Associates or technical	13	25				
Bachelor's degree	45	85				
Graduate or professional	27	48				
Income (*n* = 324)						
Less than $100,000	45	67	2.575	2	0.276	0.089^c^
$100,000–$199,000	41	84				
$200,000 or more	26	61				
Relationship status (*n* = 326)						
Single, never married	13	21	6.804	4	0.147	0.144^b^
Living with partner	5	4				
Married	80	176				
Divorced/Separated	9	8				
Widowed	5	5				
Children in home (*n* = 330)						
Yes	56	100	0.143	1	0.705	0.021^a^
No	59	115				
Industry role (*n* = 330)						
Farm owner	86	174	1.791	2	0.408	0.074^b^
Farm manager	21	31				
Farmworker	8	10				
Unhappy in role (*n* = 330)						
Never or rarely	34	166	71.235	1	< 0.001^***^	0.465^a^
Frequently	81	49				
Generation (*n* = 330)						
First-gen	31	66	0.505	1	0.477	0.039^a^
Generational	84	149				
Farm type (*n* = 325)						
Small family	43	122	13.213	3	0.004^**^	0.202^b^
Midsize family	22	31				
Large family	36	40				
Non-family	14	17				
Living arrangements (*n* = 329)						
On-farm	64	149	6.399	1	0.011^*^	0.139^a^
Off farm	51	65				
Income source (*n* = 330)						
Farm-only income	43	46	9.733	1	0.002^**^	0.172^a^
Other	72	169				
Working hours (*n* = 321)						
0–40 h	20	62	6.481	3	0.090	0.142^c^
41–60 h	35	66				
61–80 h	38	54				
81 or more h	19	27				

Sadness and depression was classified as a “yes” if participants indicated they felt sad or depressed at least once per month within the past 3 months (i.e., at least once per month, at least once per week, or daily was selected). Categorical variables were analyzed using Chi-Square.

For effect size: ^a^Phi reported. ^b^ Cramer's V reported. ^c^ Eta reported.

^*^, ^**^, ^***^ denotes significance at 0.05, 0.01, and 0.001 levels, respectively.

#### 4.4.3 Objective 3c: predict sadness and depression

We used these findings to inform the model development for our binary logistic regression to determine the effects of relevant predictors on the likelihood that Florida farmers would experience feelings of sadness and depression. We included job satisfaction, farm type, living arrangements, and income source because of their earlier bivariate significance. Primary farm role and perceived stress were included based on the precedent in existing literature, relevance to study objectives, and the theoretical relevance of stress on long-term mental health outcomes as proposed by Lazarus (30). We used a Box-Tidwell test to check the assumption of linearity for continuous predictors. The interaction term for perceived stress (*p* = 0.849) was insignificant. VIF values (all < 2) suggested no significant signs of multicollinearity. The logistic regression model using farmers' (*n* = 305) perceived stress, job satisfaction, farm type, living arrangements, and income source as explained predictors of sadness and depression, was statistically significant, χ^2^(9) = 136.756, *p* < 0.001. The model explained 49.4.% (Nagelkerke's *R*^2^) of the variance in feelings of sadness and depression in Florida farmers (*n* = 305) and correctly classified 77.0% of cases (see Table 11). Job satisfaction (*p* < 0.001), and perceived stress (*p* < 0.001) predicted sadness and depression in our model.

**Table 11:** Regression coefficients from the binary logistic regression model predicting sadness and depression in Florida farmers (*n* = 305).

Variable	*B* (SE)	df	*p*-Value	Exp(B)
Intercept	−5.249 (0.741)	1	< 0.001	0.005
Industry role		2	0.115	
Farm manager/supervisor	−0.977 (0.470)	1	0.037^*^	0.376
Farmworker	−0.294 (0.671)	1	0.662	0.745
Job satisfaction (Unhappy in role)	1.353 (0.326)	1	< 0.001^***^	3.869
Farm type		3	0.877	
Midsize family	0.171 (0.454)	1	0.706	1.187
Large family	0.257 (0.4445)	1	0.563	1.294
Non-family	0.473 (0.607)	1	0.436	1.605
Living arrangements (On-farm)	−0.456 (0.344)	1	0.185	0.634
Income source (Farm only)	−0.016 (0.387)	1	0.968	0.984
Perceived stress	0.175 (0.027)	1	< 0.001^***^	1.192

Dependent (0 = Not, 1 = Sad/Depressed) is experiencing feelings of sadness or depression, so coefficients represent likelihood of experiencing sadness or depression. The reference group for primary farm role was farm owner. The reference group for farm type was small family.

^***^ denotes significance at 0.001 level.

^*^denote significance at 0.05 level.

Every unit increase in perceived stress increased the odds of reporting feelings of sadness or depression by 1.192 (*p* < 0.001); and those who were unhappy in their role were 3.869 times more likely to report feelings of sadness or depression (*p* < 0.001), all other things held equal. Additionally, while the effect of industry role as an overall variable was not significant, results suggested a significant effect for farm managers and supervisors (Exp(*B*) = 0.376, *p* = 0.037), which indicated managers and supervisors were 62.4% less likely than farm owners to report feelings of sadness and depression, all other variables held constant.

### 4.5 Suicide ideation among Florida farmers

#### 4.5.1 Objective 4a: describe suicide ideation

Suicide ideation was the least prevalent (*n* = 330, *M* = 0.15, *SD* = 0.50), though not non-existent, in our sample. Of item respondents, 10.3% (*f* = 34) reported suicide ideation in the past 3 months. Of those who had thoughts of suicide, 7.9% (*f* = 26) had thoughts of dying by suicide less than once per month, meaning 2.4% (*f* = 8) reported having suicidal thoughts once a month or more (see Table 9). These item responses were transformed into event counts for a binary variable. Those who indicated that at any point in the past 3 months they had thoughts of dying by suicide were coded as a “Yes” (*n* = 34). All others were coded as a “No” (*n* = 296).

#### 4.5.2 Objective 4b: identify significant differences in suicide ideation

We aimed to identify if there were significant differences in suicide ideation based on personal demographics and farm-related factors. Chi-square analyses identified significant relationships between suicide ideation and age [χ^2^ (3, *N* = 190) = 9.431 *p* = 0.024, ϕ = 0.223], relationship status [χ^2^ (1, *N* = 326) = 5.161, *p* = 0.023, ϕ = 0.126], and job satisfaction [χ^2^ (1, *N* = 330) = 15.449, *p* < 0.001, ϕ = 0.216; see Table 12]. The lack of relationships in other areas is likely due to the low prevalence of suicide ideation in our sample. However, we do offer a breakdown of contingency table results to provide insight into suicide ideation in our sample.

**Table 12:** Differences in suicide ideation in Florida farmers based on demographic and farming-related factors.

Variable	*N*		*χ^2^*	df	*p-*Value	ϕ
	Yes (*n* = 34)	No (*n* = 296)				
Gender (*n* = 328)						
Male	22	186	0.167	1	0.683	0.023^a^
Female	11	109				
Age (*n* = 190)						
18–34 years	5	29	9.431	3	0.024^*^	0.223^b^
35–54 years	6	72				
55–74 years	2	65				
75 and older	3	8				
Education (*n* = 329)						
HS, up to some college	5	81	3.459	3	0.326	0.103^b^
Associates or technical	6	32				
Bachelor's degree	15	115				
Graduate or professional	7	68				
Income (*n* = 324)						
Less than $100,000	45	67	2.575	2	0.276	0.089^b^
$100,000–$199,000	41	84				
$200,000 or more	26	61				
Relationship status (*n* = 326)						
Married or with partner	22	243	5.161	1	0.023^*^	−0.126^a^
Other	11	50				
Children (*n* = 330)						
Yes	12	144	2.182	1	0.140	−0.081^a^
No	22	152				
Industry role (*n* = 330)						
Farm owner	25	235	2.025	2	0.363	0.078^b^
Farm manager	8	44				
Farmworker	1	17				
Unhappy in role (*n* = 330)						
Never or rarely	10	190	15.449	1	< 0.001^***^	0.216^a^
Frequently	24	106				
Generation (*n* = 330)						
First-Gen	10	87	0.000	1	0.998	0.000^a^
Generational	24	209				
Living arrangements (*n* = 329)						
On-Farm	21	192	0.147	1	0.701	−0.021^a^
Off farm	13	103				
Farm type (*n* = 325)						
Small family	12	153	5.623	3	0.131	0.132^b^
Midsize family	8	45				
Large family	8	68				
Non-family	6	25				
Income source (*n* = 330)						
Farm only	11	78	0.558	1	0.455	0.041^a^
Other	23	218				
Working hours (*n* = 321)						
Part to full (0–40 h)	5	77	2.349	1	0.125	0.086^a^
Overtime (41+ h)	29	210				

Suicide ideation was classified as a “yes” if participants indicated they had thoughts of dying by suicide at least once within the past 3 months (i.e., any response but “Never” was selected). Categorical variables were analyzed using Chi-Square, where statistically appropriate.

For effect size: ^a^ Phi reported.

^b^Cramer's V reported.

^*^,^**^,^***^ denotes significance at 0.05, 0.01, and 0.001 levels, respectively.

#### 4.5.3 Objective 4c: predict suicide ideation

We used these findings to inform the model development for our binary logistic regression to determine the effects of relevant predictors on the likelihood that Florida farmers would experience suicide ideation. We included age, relationship status, and job satisfaction because of their earlier bivariate significance. Perceived stress was included based on the precedent in existing literature, relevance to study objectives, and the theoretical relevance of stress on long-term mental health outcomes as proposed by Lazarus (30). We used the Box-Tidwell test to check the assumption of linearity for continuous predictors. The interaction term for perceived stress (*p* = 0.923) was insignificant. VIF values (all < 2) suggested no significant signs of multicollinearity. Our logistic regression model using farmers' (*n* = 246) perceived stress, age, relationship status, and job satisfaction as explained predictors of suicide ideation was statistically significant, χ^2^(6) = 39.642, *p* < 0.001. The model explained 34.5% (Nagelkerke's s *R*^2^) of the variance in farmers' suicide ideation.

In our model, age (*p* = 0.044), relationship status (*p* = 0.003), and perceived stress (*p* < 0.001) predicted suicide ideation. In our model, all other things held constant: every unit increase in perceived stress increased the odds of reporting suicide ideation by 1.173 (*p* < 0.001); those who were 18–34 (*p* = 0.009), 35–54 (*p* = 0.017), and 55–74 (*p* = 0.007) had significantly lower odds of reporting suicide ideation, relative to those 75 and older. Being 18–34 years of age decreased the odds of reporting suicide ideation by 95% (Exp(B) = 0.05, *p* = 0.009), being 35–54 decreased those odds by 91.8% (Exp(B) = 0.082, *p* = 0.017); and being 55–74 years old decreased the odds of suicide ideation by 96.4% (Exp(B) = 0.036, *p* = 0.017). Finally, those who were married or lived with a partner were 81.9% less likely (Exp(B) = 0.181, *p* = 0.003) than all others (e.g., single, divorced, widowed) to report suicide ideation. Job satisfaction did not have a significant effect (see Table 13).

**Table 13:** Regression coefficients from the binary logistic regression model predicting suicide ideation in Florida farmers (*n* = 246).

Variable	*B* (SE)	df	*p*-Value	Exp (*B*)
Age		3	0.044^*^	
18–34 years	−2.997 (1.148)	1	0.009^**^	0.050
35–54 years	−2.504 (1.053)	1	0.017^*^	0.082
55–74 years	−3.333 (1.244)	1	0.007^**^	0.036
Relationship (Married, Partner)	−1.709 (0.583)	1	0.003^**^	0.181
Job satisfaction (Unhappy in role)	0.395 (0.615)	1	0.520	1.485
Perceived stress	0.159 (0.046)	1	< 0.001^***^	1.173

Dependent (0 = Not, 1 = Suicide Ideation) is experiencing thoughts of dying by suicide, so coefficients represent likelihood of experiencing thoughts of dying by suicide. The reference group for age was 75 and older. The reference group for relationship status was all others (single, divorced, or widowed).

^***^Denotes significance at 0.001 level.

^**^Denotes significance at 0.01 level.

^*^Denotes significance at 0.05 level.

## 5 Discussion

We examined moderate levels of stress and a variety of risk factors, aligning with previous literature and theory (20, 42). Lazarus (30) explained the impact of factors like one's environment and ability to cope on stress. Our findings highlight personal and role-related factors contributing to stress, sadness and depression, and suicide ideation that could impact Florida farmers' ability to effectively manage stress and prevent future more severe mental health outcomes.

We identified significantly higher stress scores among farmers who were: women, 18–34 years old, unmarried, non-owners, on larger operations, relying solely on farm income, living off the farm, working overtime, and who were frequently unhappy in their role. Age, relationship status, income source, job satisfaction, and weekly hours worked predicted stress among Florida farmers.

### 5.1 Personal risk and protective factors for stress, sadness and depression, and suicide ideation

In our study, stress was higher among women and those who were unmarried or without a partner, both of which generally align with literature. While recent, related studies posited gender was insignificant (53), general-population literature supports that women experience higher levels of stress than men (4). Currently, much of the focus for intervention and messaging on farm stress has been targeted toward males. Those interventions targeting females often assume a farm spouse lens. Given the rise in female farm ownership and production in Florida (6) our findings support the need for more messaging and programming focused on helping female farmers manage stress. In our study, this would be especially true for younger, female farmers with children and/or those who might rely primarily on farm income to support their family, given those significant differences identified. Other research supports the idea that off-farm income streams, especially for women, could “insulate [farmers] from some of the financial stress that some farmers experience” [(54), p. 11].

Another potential risk factor we identified for agriculturalists in our sample was relationship status, especially for those who were divorced or separated. Significant differences in stress scores suggest those who are divorced exhibited significantly higher stress than their married counterparts. Relationship status also predicted suicide ideation in our sample, where being married was protective. Previous research supports this; citing being unmarried as a risk factor for depressive symptoms among farmers (55).

At large, the narrative of the divorced or separated farmer is less visible in literature or lay media, which could likely be attributed to the promotion of traditional norms around family in the agricultural industry. Scholarship often focuses on family conflict and stress in farm couples (56) and fewer consider the impact of divorce on stress. Haugen and Brandth (57) revealed that farm families experiencing relationship problems often will not disclose or discuss their problems out of fear of stigmatization or gossip, which creates isolation and insulates them from community support.

While social ramifications are a factor of concern, divorce or separation also causes significant practical consequences for farm couples (57). Links between relationship status and finances could also explain elevated stress and ideation risk for those who are unmarried. Divorce creates additional stress and financial strain for farm families, especially when it requires separation of assets or restructuring of farm ownership (58). In our study, 48.8% of farmers indicated they were worried at least once a month or more about losing a significant amount of income. Considering trends of rising divorce rates in the United States (59), coupled with financial stressors already concerning farmers—as seen in our sample and previous studies (2), this group should not be overlooked. Additionally, farmers who are unmarried and relying on a single income may bear more financial risk or burden. Further, there are links between farming, loneliness, and mental health due to the isolating nature of the work (60). For unmarried farmers, these feelings could be exacerbated.

Additionally, we identified a significant, negative relationship between age and stress, but higher odds of reporting suicide ideation as Florida farmers aged. The significant, negative relationship we identified aligns with recent studies revealing that individuals aged 18–34 and 35–44 reported experiencing more stress than those in other age categories (4). Additionally, it supports aging theories which suggest older adults demonstrate greater ability to cope, specifically through engaging in more health-protective behaviors to mitigate stress, than young people (61). In farming populations specifically, research echoes concerning rates of stress, anxiety, and depression among younger generations (53). Alternatively, suicide ideation in our sample was higher among seniors, specifically those 75 and older. This tends to align with previous research showing among farmers, those 65 and older experience higher rates of suicide (27, 62). From our findings and previous literature, we see a complicated nuance where stress, aging, and suicide ideation intersect, and in some ways appear to be at odds. This finding is particularly concerning, as it sheds light on the relationship between the chronic impacts of stress as we age and highlights a need to more acutely examine the pipeline from stress to more severe mental health outcomes (e.g., suicide ideation and completion) in Florida farmers. At 58.9, the average age of a Florida producer (6) is slightly higher than the national average of 57.5 (13). Efforts to both prevent stress in younger farmers and support mental health in older farmers will be crucial for supporting the next generation of Florida farmers and ensuring viability of the workforce long term.

### 5.2 Work and role-related risk and protective factors for stress, sadness and depression, and suicide ideation

Perhaps more revealing was the prominent relationships between role-related factors and stress, sadness and depression, and suicide ideation. This indicates that while demographics and other sociocultural factors contribute to stress and more severe negative mental health outcomes, Florida farmers experienced significant work-related stress.

Income source predicted stress in our sample, indicating those with farm-only income were vulnerable to stress. Additionally, we identified those on small family operations reported significantly lower stress than those on larger and non-family farms. Financial concerns are a widely cited stressor for farmers and farm families. These findings align with research that suggests larger farms are more vulnerable financially (63), and others that recognize financial insecurity as a key stressor for farmers (2) especially those who rely solely on farm income. Our findings indicate additional income streams could be protective against stress for Florida farmers.

Regarding role, farm employees experienced elevated stress, comparative to owners. Literature on role differences is sparse, but research consistently supports the risk and vulnerability of farmworkers and seasonal farm employees to stress, suicide, and depression (28). Farmworkers represented a smaller percentage of our sample. Given their contribution to the state's agricultural industry (64), future iterations should increase targeted recruitment of this population to ensure wider generalizability of findings to farmworkers, specifically.

Weekly hours worked was also an identified risk factor. In our study, farmers working considerable overtime experienced heightened levels of stress. Previous research, which has identified positive correlations between stress and work hours in young farmers, supports this (65). Occupational health research also recognizes relationships between stress and injury on the job, citing long working hours, sleep deprivation and fatigue as risk factors (66). Similarly, stress and job satisfaction both predicted sadness and depression and suicide ideation in our study.

Collectively, these findings support existing literature, which indicates that a unique complication threatening farmer mental health is the inability to detach from work, the financial stress farmers face, and the growing pressures of the job (53). For farmers, these findings are of particular concern because of the often-blurred lines between work and life. Past literature recognizes farming as an occupation that is not only a job, but a lifestyle that is also central to farmers' sense of self and social identity (67). Given this, it is important that interventions account for this unique, often unavoidable dynamic and promote reasonable work-life integration. Further, it underscores the need to invest in policies and preventative programs that target work-related stressors, reduce financial burdens, and improve overall job satisfaction among Florida farmers. In the wake of agricultural market volatilities, rising input costs, and trends forecasting continued declines in the number of producers and farms nationally (13, 68), these efforts will be critical to recruit and retain a healthy, sustainable agricultural workforce.

Farmers in our sample indicated they had thoughts of dying by suicide in the past 3 months, though ideation was lower in our study in comparison to similar studies in Georgia, USA (20, 42), Brazil (28), and the United Kingdom (69). Given the potentially sensitive nature of the topic and continued stigma around suicide, it is possible that suicide ideation was underreported and social desirability bias was present—a common limitation in suicidal behavior research (70). Given the low prevalence of suicide ideation in our sample, generalizations should be made with caution. However, results support continued monitoring of farmers' suicide risk and mortality.

Relative to suicide ideation; however, we did identify higher rates of negative emotions like anxiousness and worry, loneliness, and sadness or depression and stress. In our study, stress often precedes more severe mental health challenges, including suicide. The conclusion that stress predicted sadness and depression and suicide ideation in our sample is also widely accepted in literature (71). Other research on farm stress echoes this relationship, citing high stress as a risk factor for depression and other negative mental health outcomes (72). Theoretically, Lazarus (30) position stress as an antecedent for more compromised mental health. The theory, and others who have used it as a frame recognize that an inability to cope with stress leads to maladaptive coping behaviors could have more widespread impact on an individual's health (30, 73). Health practitioners cite connections between mental and physical health and the negative impacts of stress on the body (74). Based on this finding linking farmer stress to sadness and depression and suicide ideation, stress prevention and effective coping could be tools to help farmers' manage work-related stress, improve their quality of life, and avoid negative, long-term health impacts.

### 5.3 Recommendations for policy

These findings highlight a pressing need to obtain state and national bipartisan support for policies that prioritize farmer health and wellbeing. The Agriculture Improvement Act of 2018, recognized by many agriculturalists as the Farm Bill, is a critical piece of comprehensive legislation that governs protections, programming, and resource access for U.S. farmers and ranchers. Through the Farm Bill, initiatives like the reauthorization of regional Farm and Ranch Stress Assistance Networks (FRSANs) address critical gaps in mental health education and resource access. While the bill is currently being supported through extensions, these findings reinforce the pressing need to ensure bipartisan support to pass a new Farm Bill expeditiously. Without updated legislation in place, farmers remain unprotected and vulnerable to risk. Where federal gaps exist, policymakers could consider passing state legislation to address local agricultural needs through financial and programmatic support. We would recommend those provisions consider farmer mental health.

This research indicates a continued need for federal and state funding for FRSANs or similar regional networks. That said, we do recommend a concerted effort to reform and streamline evidence-based programming for stress prevention and coping across the Southern region. Further, this study highlights the importance of retaining and expanding health-related provisions in the Farm Bill and other state and federal legislation. Through these supports, farmers can experience increased access to mental healthcare, education, and regional resources to reduce suicide risk and mortality (29).

### 5.4 Recommendations for practice and future research

Up until this point, much of the focus nationally, regionally through FRSANs and statewide, has centered on crisis response and suicide prevention for farmers (75). However, our findings indicate Florida should consider prioritizing prevention and stress management, rather than crisis response. We recommend future resources, interventions, education, and messaging be designed to address farmer mental health using a systems-based approach. Prevention efforts should promote whole farmer health and consider mitigation and management of both personal and work-related stressors. Additionally, our findings suggest targeted efforts to support niche populations, and diverse audiences could be worthwhile, perhaps through social (76–78) or cohort-based models.

While these would be relevant across the industry, early prevention programming and resources might target women, younger farmers, and those who are unmarried—perhaps those already engaged in audience-segmented programming like the USDA Women in Agriculture Mentoring Network or the American Farm Bureau Federations' (AFBF) Young Farmers and Ranchers Program. Programming could focus on stress management and coping and work-life integration to continue fostering job satisfaction among Florida's agricultural workforce. Research should continue to explore stress in female farmers and the impact of stress on the individual, family, and business levels. Similarly, it will be important to continue to monitor the social, emotional, and financial impacts of divorce on stress in farmers and farm families. Additionally, while a less represented group in our study, farmworkers continue to be an underserved population. Our findings indicate that farm employees experienced heightened levels of stress. Future studies examining stressors by role type could help magnify sources of stress for targeted policies and interventions. Moreover, research to examine audience segmentation and effective communication strategies for reaching these diverse audiences in agriculture would be valuable.

This collaborative, industry-supported study expanded on pilot testing in Georgia (20) and was the first of its kind in Florida. We recommend scaling this study for regional and national replication to further validate this instrument and create a consistent measure for obtaining comparative baseline and longitudinal data. Paired with secondary analysis of state-by-state mortality data, specifically for farming occupations, future studies could garner a national scope of mental illness, suicide risk, and mortality among farmers.

Further, this study prioritized interdisciplinary partnerships across academia and the agricultural sector to provide a baseline for longitudinal stress and mental health assessment statewide. Future replications should adopt a similar community partnership model to reinforce trust and feedback loops with farmers to ensure recommendations meet local needs. If replicated regionally, data could identify stress and farmer suicide indicators in the Southeast to offer relevant message frames for agricultural communicators and industry stakeholders promoting mental health. Researchers should consider exploring seasonality and its impact on farmers' stress and coping to not only identify trends in health, but to also better understand when farmers might have the most ability or capacity to engage. Longitudinal data could inform critical intervention points for Extension educators and additional policy recommendations.

Finally, quantitative research offers a valuable, but limited lens for social behavioral research (79). Future research should capitalize on mixed methods approaches to more fully understand farmers' stress and coping, health outcomes, and intent or ability to keep farming. From a systems-perspective, future studies should consider the economic consequences of stress and mental illness on the state's agricultural industry and explore how these factors influence labor retention, the survival of generational family farms, and agricultural production long term.

### 5.5 Strengths and limitations

We adhered to Dillman et al.'s (36) tailored design method for reducing measurement error and increasing response rates. We made multiple efforts to limit non-response bias including using clear, straightforward wording of items, creating multiple contacts during recruitment, reviewing the survey with industry stakeholders for relevance, and offering a dual language version to ensure the survey is inclusive to Spanish speakers (36). We also offered small incentives during in-person recruitment. Finally, given the nature of our survey, we reiterated confidentiality before potentially sensitive items (36) and leveraged trusted industry stakeholders to promote this messaging. Given these efforts, demographics in our sample generally reflected Florida's agricultural industry (6). While the age, race, and gender of our sample was generally in line with the demographic makeup of Florida producers, farmworkers were largely underrepresented in our study (6).

Additionally, due to the nature of our sampling approaches, we were unable to report a true response rate, which is a known limitation in our study, and commonly cited challenge when using social media and venue-based approaches to recruit participants (37, 80). These approaches introduce the potential for selection bias and limit generalizability of our findings. We also recognize that not calculating a required sample size or conducting power analyses *a priori* further limits interpretation. These analyses were intentionally exploratory within the scope of the larger study to inform future research inquiry and hypothesis testing. Additionally, despite efforts to ensure confidentiality, we recognize some level of social desirability bias could be at play, as is common in self-reported survey research, especially in studies with perceivably sensitive topics like mental health and health service utilization (81). Finally, our study was cross-sectional and descriptive, and somewhat exploratory in nature. While our analyses capture potential unique relationships, they do not fully capture the complex, multifaceted interactions between antecedent factors, stress, and sadness or depression, or suicide ideation. Future, longitudinal studies should be done to accommodate more robust hypothesis and model testing. Given these limitations, we do express caution extrapolating our findings beyond this study. However, this study was the first of its kind to offer farmer-specific recommendations in Florida and could be used as a model for future replications and related studies in similar states.

## 6 Conclusions

This cross-sectional study focused on stress appraisal and potential antecedents that could be influencing Florida farmers' mental health. Stress and negative mental health outcomes continue to be of concern for Florida farmers and should continue to be monitored. We identified moderate levels of stress; and while not non-existent, suicide ideation remained less prevalent in our sample than other negative emotions like anxiousness and sadness and depression. The transactional theory of stress and coping posits that a multitude of factors influence stressors, primary stress appraisal, and stress outcomes (30, 73).

## Data Availability

The datasets presented in this article are not readily available because datasets were generated under a contracted data use agreement with the funding non-profit and community partners. However, there is potential for data to be made available by the authors, upon request and consideration. Requests to access the datasets should be directed to sab5271@ufl.edu.
