# Supplementary material for: Identifying risk and protective factors for mental health outcomes: a cross-sectional survey predicting stress, depression, and suicide ideation among Florida farmers
Source: Front Public Health. 2026 Jun 29;14:1832179. doi: 10.3389/fpubh.2026.1832179 (PMC13365348; doi:10.3389/fpubh.2026.1832179)
Supplement: Supplementary file 1 [file Image_1.pdf]

Supplementary Material

1 Supplementary Figure

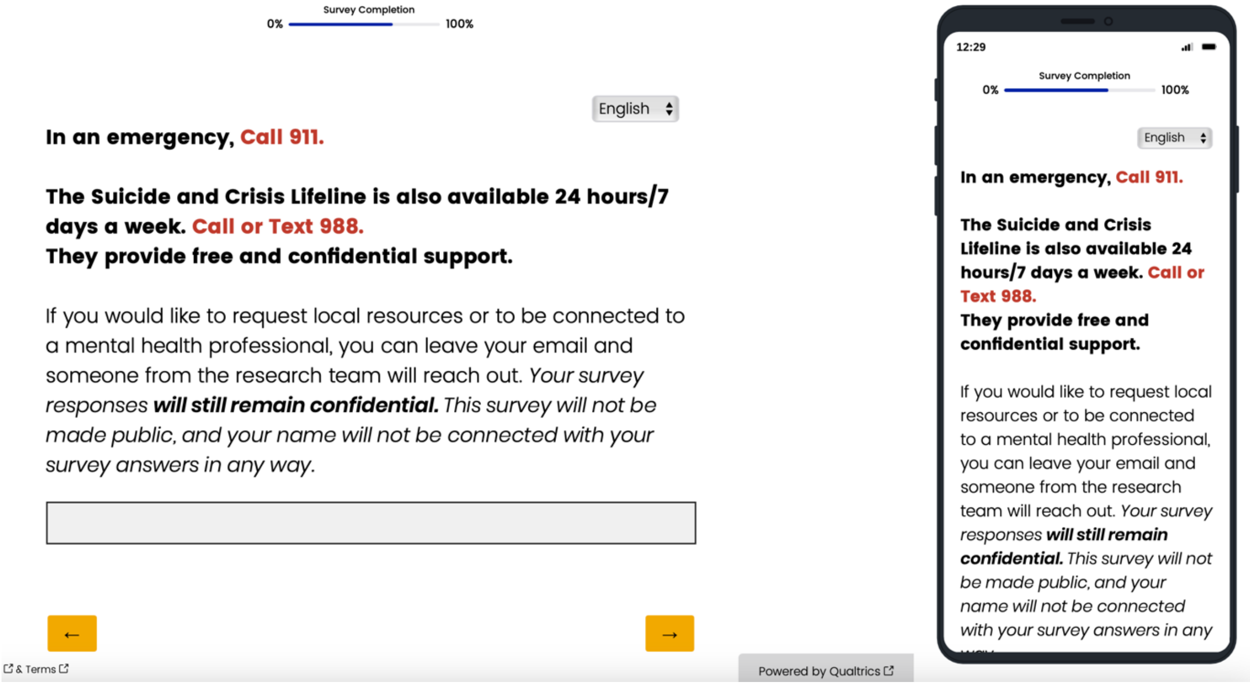

Supplementary Figure 1. Screenshot of 911 and Suicide Hotline PSA in survey - A 911 and Suicide Hotline PSA as well as a text-entry to request contact with a member of the research team was displayed after: Feeling sad or depression (IF: *at least once per week* or *daily* was selected); Feeling hopeless or like you will never be happy (IF: *at least once per month*, *at least once per week* or *daily* was selected); or Thoughts of wanting to die by suicide (IF: any item except for *never* was selected).
